# Supplementary material for: A novel gene REPTOR2 activates the autophagic degradation of wing disc in pea aphid
Source: eLife. 2023 Mar 21;12:e83023. doi: 10.7554/eLife.83023 (PMC10030113; doi:10.7554/eLife.83023)
Supplement: Supplementary file 1. [file elife-83023-supp1.docx]

**Supplementary File 1. Primers used for the study**

| Name | Orientation | Primer Sequence (5’-3’) | Accession No. | Source |
| --- | --- | --- | --- | --- |
| *ATG1a* | Forward | TGCATTGTTGAATGTGCTTCTCC | ACYPI000902 | (Simonet et al., 2018) |
|  | Reverse | CAGCTTTGAGTTCAGACGTAGC |  |  |
| *ATG1b* | Forward | AGCTAATGCTGTTGAGATTGCAC | ACYPI008229 | (Simonet et al., 2018) |
|  | Reverse | GGATGCCCGGAGGTTCTTTT |  |  |
| *ATG3a* | Forward | CTGACCCTCAGCCGAATTGT | ACYPI072921 | (Simonet et al., 2018) |
|  | Reverse | TGCACTACACAGAAACGATAAACG |  |  |
| *ATG3b* | Forward | CAAGTTTCCAACATTGTGACCCA | ACYPI001070 | (Simonet et al., 2018) |
|  | Reverse | GCATACCACTTTCTACAAACGCA |  |  |
| *ATG4* | Forward | ACTGTGATGGAACAAACTAACTCT | ACYPI001437 | (Simonet et al., 2018) |
|  | Reverse | ACACCTTGAACATAAGCAGGG |  |  |
| *ATG5* | Forward | ATGGAACCATCTGAAGGCAGT | ACYPI005861 | (Simonet et al., 2018) |
|  | Reverse | TTCAGTTTCATCAGCTCTCCCA |  |  |
| *ATG7* | Forward | TCGTAGATAAGACACTTGATCAGC | ACYPI008937 | (Simonet et al., 2018) |
|  | Reverse | TGAGACAAATAACTCGACAGCC |  |  |
| *ATG8* | Forward | AGAAGGCACCTAAATCGCGT | ACYPI004701 | (Simonet et al., 2018) |
|  | Reverse | CTTCGGGACGTAAGTGGACG |  |  |
| *ATG10* | Forward | GTTCAATCCCCTAATAGCATGGTTA | ACYPI53898 | (Simonet et al., 2018) |
|  | Reverse | ACTTTTGAGCCTTCGGTTTCCA |  |  |
| *ATG12* | Forward | TATTGAAAGCAACTGGTAATGCACC | ACYPI000775 | (Simonet et al., 2018) |
|  | Reverse | CCGTAAGAATTCATGGATAGAAGCC |  |  |
| *ATG13* | Forward | AGCCCAGAAAACGAGCTGAA | ACYPI41944 | (Simonet et al., 2018) |
|  | Reverse | GCCACAGTGTTGCTTAGTGC |  |  |
| *ATG14* | Forward | ACCCACTTGAATGCCAACAT | ACYPI005194 | (Simonet et al., 2018) |
|  | Reverse | ACAGATGTATCCAAAAGTTGCAG |  |  |
| *ATG16* | Forward | AGTTTGCCATGTCCAGTTGC | ACYPI065154 | (Simonet et al., 2018) |
|  | Reverse | TCCTCGTATCCCAGAACCGT |  |  |
| *Ark* | Forward | TCGAAGGCTTGGATCAATGGT | ACYPI49157 | (Simonet et al., 2018) |
|  | Reverse | TGCAATTTCAGACTGTACACGG |  |  |
| *Dronc1* | Forward | ACTGCAATACGTGATCCAATAGA | ACYPI25243 | (Simonet et al., 2018) |
|  | Reverse | CATCAATCTAGCCAATTCGGTATTA |  |  |
| *Dronc2* | Forward | GGCATACTGCTAAACGTGATCC | ACYPI43249 | (Simonet et al., 2018) |
|  | Reverse | TGCATTCAATTCAGTGTTATAGGCA |  |  |
| *DrICE* | Forward | GCAACTCCGTAACTACAAACTGC | ACYPI001931 | (Simonet et al., 2018) |
|  | Reverse | AAAAGCGTCGGCAGTGTCA |  |  |
| *Decay1* | Forward | TGAAAAGTGATATTGTTGTCGCA | ACYPI003094 | (Simonet et al., 2018) |
|  | Reverse | TGTGCTAAACTCAAAAGCAACA |  |  |
| *Decay2* | Forward | ATTGTTGTGTTGACACACGGG | ACYPI004566 | (Simonet et al., 2018) |
|  | Reverse | AGTTTAGGTTTTCCGGCTAGTGT |  |  |
| *IAP1* | Forward | CCAAGGTCTGATGGATTGGGA | ACYPI009246 | (Simonet et al., 2018) |
|  | Reverse | TTCACCACCAACCTCTTCCAC |  |  |
| *IAP2* | Forward | CCCTTGTCTTCACACCATTGC | ACYPI004833 | (Simonet et al., 2018) |
|  | Reverse | TCTTTGACGTTTTCTTCGTCCA |  |  |
| *IAP3* | Forward | GTTCCCGATTCGATGTTGTGC | ACYPI088105 | (Simonet et al., 2018) |
|  | Reverse | CACACAGCACACTGTTCGAG |  |  |
| *IAP4* | Forward | CGTCGTTTGAAAGAAGCCCG | ACYPI000445 | (Simonet et al., 2018) |
|  | Reverse | AGTAGCCTTGATGGTCTGTCG |  |  |
| *Deterin1* | Forward | GAACCCCTTCAACACATCTGG | ACYPI000677 | (Simonet et al., 2018) |
|  | Reverse | CTGCTTCGGCCATATCCTTG |  |  |
| *Deterin2* | Forward | AAATGGCCGAAGCAGGTTTTT | ACYPI21920 | (Simonet et al., 2018) |
|  | Reverse | AAGGCTGGTCAGTTGGCTC |  |  |
| *REPTOR1* | Forward | TGACACCAGTCAGCGAGTTG | [ACYPI008922](http://bipaa.genouest.org/apps/grs-2.3/grs?reportID=aphidbase_transcript_report&objectID=ACYPI008922) | This study |
|  | Reverse | TCACTCGTTGTTCTAGCGACT |  |  |
| *REPTOR2* | Forward | GGCCCGCTGAGCTTACAAT | ACYPI001165 | This study |
|  | Reverse | TTATCACCACCACAGGCATTT |  |  |
| *TOR* | Forward | TGGCTGTTCTTGAGGCTTTT | ACYPI004568 | This study |
|  | Reverse | CAGTTGCAGCTGCTGTTCTC |  |  |
| *actin* | Forward | AGCTCTATTCCAACCTTCCTTCT | ACYPI000064 | (Simonet et al., 2018) |
|  | Reverse | TGTATGTAGTCTCGTGGATACCG |  |  |
| *dsREPTOR1* |  | CTAGCCCTGTTAACCGACCA |  | This study |
|  |  | CTGTCGAGGTTCTCTACGC |  |  |
|  |  | T7-CTAGCCCTGTTAACCGACCA |  |  |
|  |  | T7-CTGTCGAGGTTCTCTACGC |  |  |
| *dsREPTOR2* |  | TGCCTGTGGTGGTGATAATTT |  | This study |
|  |  | TGGTGAATGTTGAAAGCCAGACT |  |  |
|  |  | T7-TGCCTGTGGTGGTGATAATTT |  |  |
|  |  | T7-TGGTGAATGTTGAAAGCCAGACT |  |  |
| *dsTOR* |  | CTGGTTGGCGAATCGTTGTG |  | This study |
|  |  | ACTTCCAACAGACCCGATGC |  |  |
|  |  | T7-CTGGTTGGCGAATCGTTGTG |  |  |
|  |  | T7-ACTTCCAACAGACCCGATGC |  |  |
| *dsGFP* |  | CTCGTGACCACCCTGACCTAC |  | This study |
|  |  | GTTCACCTTGATGCCGTTCTT |  |  |
|  |  | T7-CTCGTGACCACCCTGACCTAC |  |  |
|  |  | T7-GTTCACCTTGATGCCGTTCTT |  |  |
| *REPTOR2* probe |  | CAUGUAGGUAUAUCACCCUCUUAAACCUUG |  | This study |
| *TOR* probe |  | CAGUCUCAAUGUUACCAAUAUCACCUCC |  | This study |

T7 promoter sequence: 5’-TAATACGACTCACTATAGG-3’
